# Investigation on the Biological Safety and Activity of a Gluconolactone-Based Lotion for Dermocosmetic Application

**DOI:** 10.3390/ph16050655

**Published:** 2023-04-27

**Authors:** Nicola Zerbinati, Serena Di Francesco, Maria Chiara Capillo, Cristina Maccario, Giorgio Stabile, Hassan Galadari, Raffaele Rauso, Sabrina Sommatis, Roberto Mocchi

**Affiliations:** 1 Department of Medicine and Surgery, University of Insubria, 21100 Varese, Italy; 2 UB-CARE S.r.l., Spin-Off University of Pavia, 27100 Pavia, Italy; 3 Department of Medicine and Surgery, University of Vita-Salute San Raffaele, 20132 Milano, Italy; 4 College of Medicine and Health Sciences, United Arab Emirates University, Al Ain P.O. Box 15551, United Arab Emirates; 5 Head and Neck Unit, Clinica Cobellis, 84078 Vallo della Lucania, Italy

**Keywords:** cosmeceutical, gluconolactone, skin care, biosafety, skin-ageing

## Abstract

(1) Background: Cosmeceuticals are formulas enriched with active ingredients that exert efficacy on different skin molecular targets. (2) Methods: Cell viability and the absence of potential irritant risk were evaluated on keratinocytes (HaCaT), fibroblasts (NHDF), adipocytes (3T3-L1), sebocytes (PCi-SEB_CAU) and reconstructed human epidermis (RHE), respectively. Several treatments were performed to evaluate the ability of the lotion to stimulate the production of collagen and elastin, stimulate the differentiation of keratinocytes and reduce the number of senescent cells following UVB stimulation. In addition, the modulation of genes involved in the production, storage and accumulation of sebum were investigated. (3) Results: The results obtained demonstrated the biosafety of the formula in all cell lines tested. The 24-h treatment with non-cytotoxic concentrations determined an increase in the expression of the collagen (COL1A1), elastin (ELN) and involucrin (IVL) genes, while a reduction of peroxisome proliferator-activated receptor-gamma (PPARγ) gene expression and a reduction of SA-βgal-positive cells were found. Moreover, the treatment did not interfere with normal steroid 5-alpha reductase (5RDA3) gene expression levels. (4) Conclusions: Data collected demonstrated the biosafety of the lotion, the non-comedogenic property and a multi targets anti-aging effect. In particular, data collected on the booster lotion make it a valid way to counteract the pore dilatation aging related.

## 1. Introduction

Human skin is a sophisticated and dynamic organ that plays a key role as a barrier between internal matter and the world outside, yet has numerous functions such as homeostatic regulation, the prevention of the percutaneous loss of fluid, electrolytes, and proteins, temperature maintenance, sensory perception and immune surveillance [1]. Human integument, which comprises one-sixth of total body weight, forms the most visible indicator of age. The synergistic effects of intrinsic and extrinsic ageing factors produce deterioration of the cutaneous barrier, wrinkles formation, skin surface roughness, skin dullness and skin laxity. In addition, aged skin is susceptible to pervasive dryness and itching, cutaneous infection, vascular complications, increased risk of cutaneous malignancy and autoimmune disorders. Recently, instances in the literature have correlated factors, such as ageing, autoimmune disorders such as thyroid disease, alopecia areata and atopic dermatitis, with vitiligo, an autoimmune depigmenting disorder of the skin [2,3]. Genetic predisposition, ethnicity and hormonal changes in cutaneous tissues are the main intrinsic factors that can compromise healthy skin. On the other hand, lifestyle influences, the effects of smoking, nicotine, pollution and exposure to UV light (photo-ageing) represent the wide range of extrinsic factors that can interfere with the skin balance [4,5]. Each of these factors can act in a targeted manner on one or more tissues or cell populations, compromising the perfect balance that determines the functionality of this organ. Among the main structural alterations that are related to ageing, the loss of the extracellular matrix structure (ECM) caused by the arrangement of collagen and elastin fibers must be considered. The loss in elasticity is due to the action of matrix metalloproteinases (MMPs) degrading the main constituents of the ECM, such as types I and III collagen, proteoglycans and glycosaminoglycans (GAGs) [6]. In particular, elastic fibers are exposed to a variety of enzymatic, chemical and biophysical influences over the lifespan of an organism and accumulate damage due to their low turnover. On the molecular level, during ageing, elastin undergoes diverse types of detrimental alterations ranging from enzymatic degradation, oxidative damage, the formation of advanced glycation end products (AGEs), calcification, aspartic acid racemization, lipid binding, carbamylation and mechanical fatigue. Reduced production or alterations of these structural proteins also negatively affect the stimulation of new cell growth by fibroblasts, which replace the dead skin cells and encourage healthy cell turnover promotion [7,8]. Regarding the maintenance of skin elasticity, some studies have been conducted to evaluate the anti-ageing effects of active ingredients in cosmetic products (e.g., soybean extract and retinol) able to stimulate the replacement of the skin’s elastic fibers, improving the elasticity of dermis equivalents in vitro, as well as skin biomechanical properties and appearance in vivo [9]. Furthermore, it has been widely recognized that with ageing the number of visible pores increases, as well as their surface and the alteration of their shape, which tends to become elliptical. In the cheeks of women over 40 years old, the skin pore walls sag and become dilated [10]. Sebum is secreted from sebaceous glands, which are composed of sebaceous follicles. Sebocytes are the predominating cells around sebaceous glands, and they express dihydrotestosterone (DHT) receptors, which influence sebaceous gland activity in terms of sebum secretion. The steroid 5-alpha reductase gene (SRD5A) plays a key role in the regulation of DHT; thus, the inhibition of its expression can decrease the amount of DHT in cells, leading to facial sebum control [11].

Excess sebum production also plays a key role in the previously mentioned parakeratosis condition, where the abnormal accelerated keratinization process leads to the accumulation of nucleated cells also involved in the dilation of pores. To restore the normal keratinization process and thus reduce the unsightly appearance of the dilated pores, it is necessary to stimulate the differentiation of the keratinocytes, favoring the renewal of the nucleated cells around the dilated pores by new mature corneocytes [12,13]. The alteration of sebum production is also one of the main factors involved in comedone formation. Generically, the term comedogenicity refers to the potential of various agents to promote the abnormal differentiation of the follicular epithelium that results in the formation of microcomedones [14]. Non-appropriate cosmetics not only aggravate or prolong pre-existing acne vulgaris, but can produce them as well, especially in adults, being a particular variant of acne, acne cosmetica. The comedone is the primary acne lesion. It results from a partial (open comedone or blackhead) or complete (closed comedone or whitehead) obstruction of the pilosebaceous duct and an accumulation of sebum [15]. Among the possible therapeutic targets for this skin disorders, particular attention has been dedicated to the role of peroxisome proliferator-activated receptors (PPARs), which are ligand-activated transcription factors that modulate the expression of multiple genes involved in the regulation of lipid, glucose and amino acid metabolism. In humans, all three PPARs isotypes are expressed in epidermis and, in particular, PPARγ is mostly found in the more differentiated suprabasal keratinocytes, in skin adipocytes and sebaceous glands (SGs), where it plays a critical role in differentiation and lipid formation. It is a master regulator of adipogenesis and lipid storage, a central player in thermogenesis and an active modulator of lipid metabolism and insulin sensitivity. Furthermore, exercise control over sebum production can lead to a reduction of oily or greasy skin, and to a normalization of the pore size [16].

Counteracting the signs of ageing, as well as correcting skin imperfections, are among the main purposes of aesthetic medicine, with the dual purpose of improving people’s compliance and, at the same time, preventing complications due to the loss of the functionality of the human skin. Moving beyond traditional cosmetics, which only temporarily adorn and beautify the skin, cosmetics companies have tapped into the biomedical revolution, adding biologically active ingredients to their facial emollient lotions and creams to enhance the function of healthy skin [17,18]. Our study presents an in vitro test panel to test the biocompatibility and efficacy of a booster lotion (PHA TOUCH, Matex Lab S.p.a, Brindisi, Italy) on more superficial (keratinocytes) and deeper (fibroblasts, adipocytes and sebocytes) cell lines. The formula contains several compounds that are highly recognized for their ability to stimulate collagen and elastin synthesis, reduce pore size and make skin texture more uniform through a non-comedogenic action and by promoting cell renewal [19,20,21]. In particular, it contains gluconolactone, a molecule that belongs to a group of poly-hydroxy acids (PHA); it has a hydroxyl group on a carbon, but it also presents other hydroxyl groups elsewhere in the carbon chain that make it an antioxidant molecule able to protect against the effects of free radicals caused by ultraviolet-B (UVB) damage [22]. These radiations, which penetrate the skin causing free radical production, ultimately damage cell membranes, enzymes and extracellular matrix proteins. Ideally, any free radical is removed from cells via enzymatic and nonenzymatic mechanisms. Antioxidant molecules scavenge free radicals and protect cells from damage, preventing UV-related photo-ageing [23]. In skin care products, gluconolactone can act as either an active ingredient (exerting benefits for the skin) or as an adjuvant (an ingredient that is included to help improve the overall quality of the product) [24]. As an active ingredient, gluconolactone is often added to formulas for its skin-conditioning properties, such as chemical exfoliating, dissolving the dead cells from the outermost layer of the skin, resulting in a smoother and brighter complexion, improving texture and tone [25]. For this molecule, the ability to remove excess oil and exert skin sebum control has also been recognized, as well as its decreased potential for unsightly side effects, such as redness and flaking, due to the large dimensions that lead to a slow penetration of this molecule in the deeper skin layers [22].

Many of its activities are also exerted by alpha hydroxy acids (AHAs), a group of organic carboxylic acids that have a hydroxy group in the alpha position. These compounds are popular skin rejuvenators and they are found in many moisturizers, cleansers and cosmetic products. Salicylic acid is the only beta hydroxy acid (BHA) that has an organic carboxylic acid with a hydroxy group in the beta position. This phenolic compound is hydrophobic and lipophilic. It can enter the sebaceous unit and it has been used for decades by dermatologists as a comedolytic [25].

Therefore, starting with the PHA TOUCH chemical composition, careful bibliographic research was conducted to select the main molecular targets to demonstrate its safety and non-comedogenic properties, its anti-ageing and photo-ageing effect, as well as its ability to stimulate cell renewal and minimize pore size, making skin texture more uniform.

## 2. Results

### 2.1. Evaluation of Cell Viability

**Figure 1:**
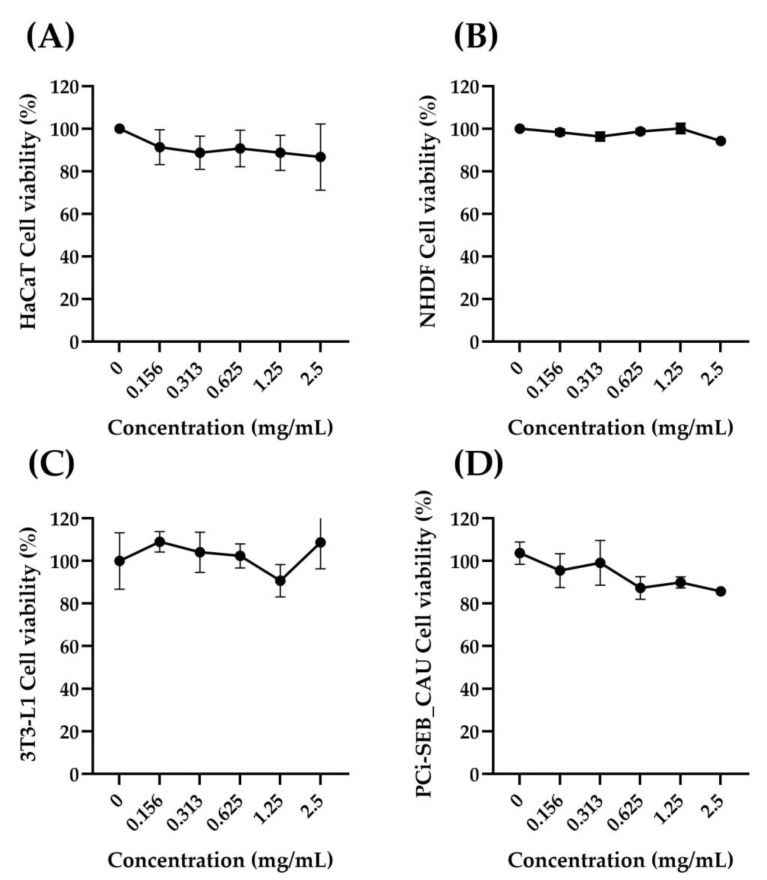
Cell viability (%) of HaCaT (**A**), NHDF (**B**), 3T3-L1 (**C**) and PCi-SEB_CAU (**D**) cell lines after treatment with different concentrations of the cosmeceutical product (range between 0.156 and 2.5 mg/mL) with respect to the control (untreated cells) (*n* = 2; replicates = 3).

A cytotoxicity test is required to evaluate the effect of the cosmeceutical on the cell viability of each cell line selected to perform the study, but also to determine the appropriate concentrations required for the following efficacy assays. Data collected show that the product does not show cytotoxic activity after 24 h of treatment with all tested concentrations (0.156–2.5 mg/mL) on human fibroblasts (NHDF), keratinocytes (HaCaT), adipocytes (3T3-L1) and sebocytes (PCi-SEB_CAU) cell lines (Figure 1).

### 2.2. Skin Irritation on Reconstructed Human Epidermis (RHE) Model

**Figure 2:**
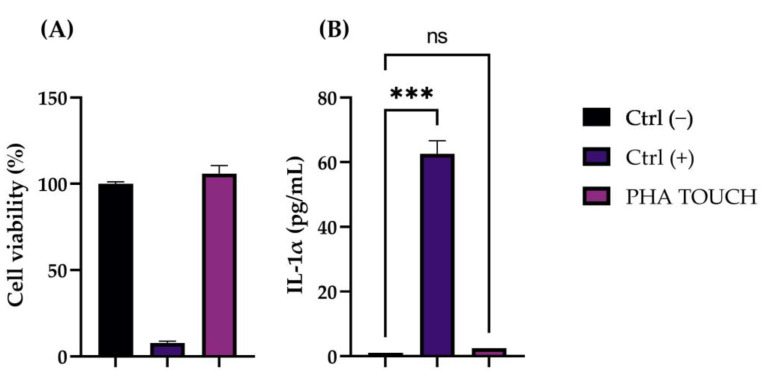
(**A**) Cell viability expressed as a percentage (%) of RHE treated with the cosmeceutical. Ctrl (−): RHE treated with Dulbecco Phosphate Buffer Solution (DPBS); Ctrl (+): RHE treated with Sodium Dodecyl Sulfate (SDS) as the irritating stimulus. (**B**) IL-1α amount (pg/mL) in the medium after treatment with the tested product; Ctrl (−): cells treated with DPBS; Ctrl (+): RHE treated with SDS. Values of *p* ≤ 0.001 (***) were considered statistically significant compared with Ctrl (−) (*n* = 1, replicates = 2).

To evaluate the safety of the cosmetic product and the absence of a potential risk of skin irritation, in accordance with the guidelines, cell viability and the IL-1α amount were analyzed using an ELISA kit were investigated on a 3D model of human epidermis (RHE) [26]. The results obtained showed a viability of 105% and an amount of interleukin IL-1α released equal to 2.52 pg/mL after treatment with the cosmeceutical on the RHE model (Figure 2), demonstrating the absence of a potential risk of irritation (threshold: viability ≥ 50%; IL-1α ≤ 10 pg/mL) [27].

### 2.3. Collagen and Elastin Synthesis

**Figure 3:**
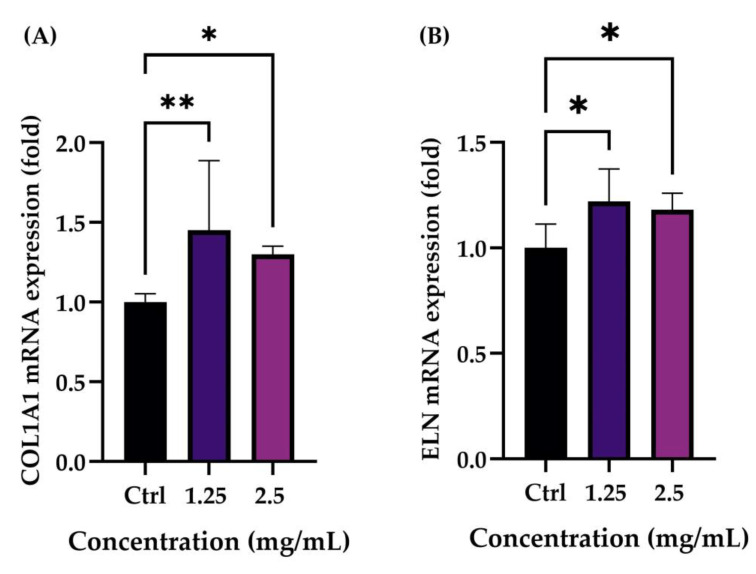
(**A**) Collagen (COL1A1) and (**B**) elastin (ELN) mRNA expression after treatment with the cosmetic product at the selected concentrations (1.25 and 2.5 mg/mL) with respect to the control (Ctrl, untreated cells) in NHDF cells. *p* ≤ 0.05 (*) and *p* ≤ 0.01 (**) were considered to be statistically significant compared with untreated cells (*n* = 2; replicates = 2).

To evaluate the possible modulation of collagen and elastin levels after treatment with the product, NHDF cells were treated with two concentrations (1.25 and 2.5 mg/mL) of the product selected by a preliminary cell viability assay. The obtained data highlighted that treatment with both tested concentrations induced an increase in collagen and elastin production compared to the control (Ctrl, untreated cells). The data were statistically significant, demonstrating the efficacy of the product to modulate the production of two of the main proteins involved in the ageing process (Figure 3).

### 2.4. Evaluation of Keratinocytes Differentiation Marker

**Figure 4:**
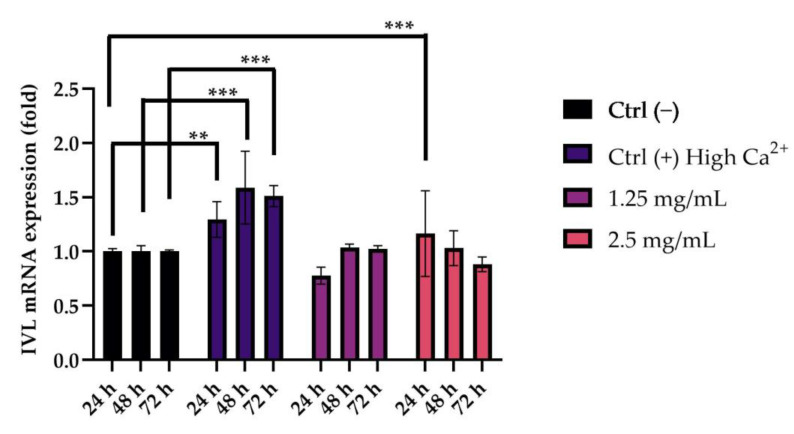
Graphical representation of IVL gene expression levels measured in mRNA expression after treatment, for different time points (24-48-72 h), with the cosmetic product at selected concentrations (1.25 and 2.5 mg/mL) in HaCaT cells. Ctrl (−): untreated cells; Ctrl (+): cells treated with high Ca^2+^ concentration. *p* ≤ 0.01 (**) and *p* ≤ 0.001 (***) were considered to be statistically significant compared with untreated cells (*n* = 2; replicates = 2).

HaCaT cells were treated at different time points (24-48-72 h) with the concentrations selected by preliminary cell viability assay (1.25–2.5 mg/mL) in order to evaluate the expression of one of the main proteins involved in the keratinocytes differentiation (involucrin, IVL). The obtained data highlighted that, after 24 h of treatment with the cosmetic product at a concentration of 2.5 mg/mL, the expression of the differentiation marker IVL significantly increased compared to the untreated cells (Ctrl −). The positive controls (Ctrl +) were incubated with high concentrations of Ca^2+^, which led to a statistically significant increase in IVL expression after all tested time points (Figure 4).

### 2.5. Evaluation of Anti Photo-Ageing in NHDF Irradiated Model

**Figure 5:**
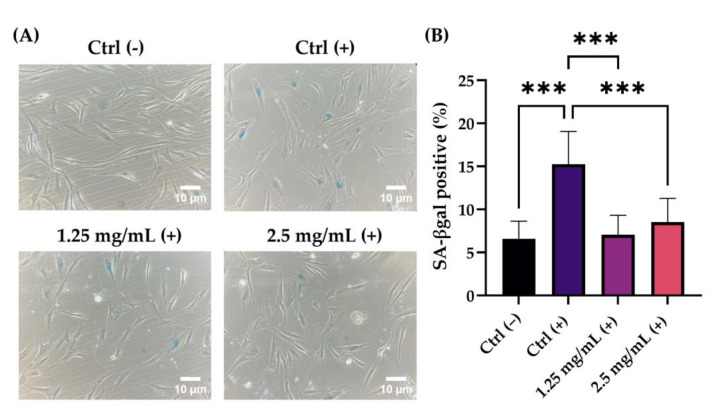
(**A**) Representative images obtained by optical microscope (20× magnification) of NHDF blue-stained cells for β-galactosidase positivity. Ctrl (−): untreated cells; Ctrl (+): untreated cells irradiated with 38 mJ/cm^2^ UVB-radiation; 1.25 mg/mL (+): NHDF treated with 1.25 mg/mL of the cosmetic product and irradiated with 38 mJ/cm^2^ of UVB-radiation; 2.5 mg/mL (+): NHDF treated with 2.5 mg/mL of the cosmetic product and irradiated with 38 mJ/cm^2^ of UVB-radiation. (**B**) Graphical representation of SA-βgal expression levels as percentage of positive cells for each tested condition. Ctrl (−): untreated cells; Ctrl (+): untreated cells irradiated with 38 mJ/cm^2^ UVB-radiation; 1.25 mg/mL (+): NHDF treated with 1.25 mg/mL of the cosmetic product and irradiated with 38 mJ/cm^2^ of UVB-radiation; 2.5 mg/mL (+): NHDF treated with 2.5 mg/mL of the cosmetic product and irradiated with 38 mJ/cm^2^ of UVB-radiation. *p* ≤ 0.001 (***) were considered statistically significant (*n* = 2; replicates = 3).

A model of stress-induced premature senescence of NHDF cells was reproduced by UVB irradiation as the source of extrinsic damage. A preliminary cytotoxicity evaluation was performed to verify that the treatment with the selected concentrations maintained good viability even in association with UVB radiation at a sub-toxic dose of 38 mJ/cm^2^. Therefore, cells were treated with 1.25 and 2.5 mg/mL of the product and irradiated with 38 mJ/cm^2^ to evaluate the potential ability to prevent cells from photo-ageing by β-galactosidase staining. The obtained results showed a significant increase in SA-βgal-positive cells in untreated but irradiated cells (Ctrl +) compared to untreated cells (Ctrl −). Moreover, the cosmeceutical product resulted in a statistically significant reduction in SA-βgal activity levels of 54% and 44%, respectively, after treatment with two selected concentrations of 1.25 and 2.5 mg/mL (Figure 5).

### 2.6. Evaluation of Efficacy in Sebum Production and Adipogenesis

#### 2.6.1. Modulation of Gene Expression

**Figure 6:**
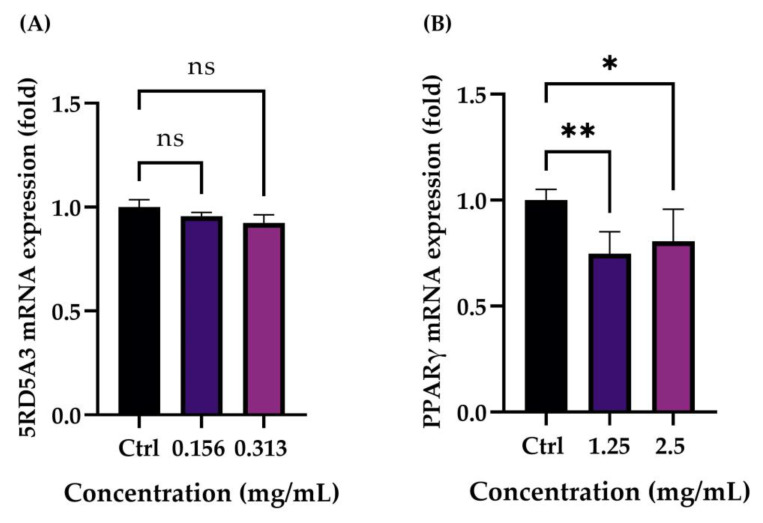
5RD5A3 (**A**) and PPARγ (**B**) mRNA expression after treatment with the cosmetic product at the selected concentrations (0.156 and 0.313 mg/mL for 5RD53A analysis in sebocytes and 1.25 and 2.5 mg/mL for PPARγ investigation in adipocytes) with respect to the relative control (Ctrl, untreated cells). *p* ≤ 0.05 (*) and *p* ≤ 0.01 (**) were considered to be statistically significant compared with the respective control (*n* = 2; replicates = 2).

In order to evaluate a possible modulation of SRD5A3 gene expression, sebocytes (PCi-SEB_CAU) were treated with the product at concentrations of 0.156 and 0.313 mg/mL, selected by a further MTT performed in a 12-well plate (on which the treatment for the analysis of SRD5A3 in RT-qPCR was performed). The data obtained demonstrated a greater sensitivity of the cell line compared to the preliminary cytotoxicity test performed on the 96-well plate (see Section 2.1). On the other side, adipocytes (3T3-L1) were treated with the selected concentration (1.25 and 2.5 mg/mL) to evaluate the PPARγ gene expression. The data obtained highlighted that treatment with the cosmeceutical at all tested concentrations does not determine a variation of SRD5A3 gene expression compared to untreated cells, proving that the product does not affect the sebaceous lipid production (Figure 6A). The results collected regarding PPARγ gene expression showed that treatment with both concentrations tested led to a statistically significant reduction in the marker compared to the untreated cells, suggesting a decrease in the adipogenesis and lipogenesis processes (Figure 6B).

#### 2.6.2. Modulation of Lipid Content by Oil Red O Staining

**Figure 7:**
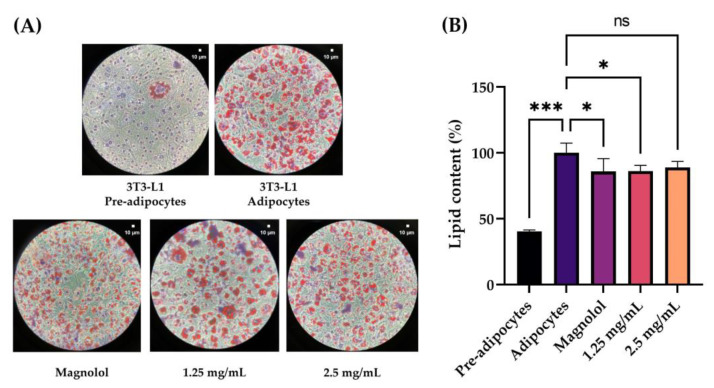
(**A**) Representative images of the lipid drops observed under an optical microscope (20× magnification) before extraction with isopropanol of 3T3-L1 cells stained with Oil Red O and Hematoxylin after 24 h of treatment with the tested conditions. (**B**) Quantification of lipid accumulation in percentage after extraction with isopropanol for each tested condition. 3T3-L1 pre-adipocytes: cell line pre-differentiation; 3T3-L1 adipocytes: cell line post-differentiation; Magnolol: 3T3-L1 adipocytes after treatment with magnolol 20 µM as the positive control; 1.25 mg/mL: 3T3-L1 adipocytes after treatment with the cosmeceutical product at 1.25 mg/mL concentration; 2.5 mg/mL: 3T3-L1 adipocytes after treatment with the cosmeceutical product at 2.5 mg/mL concentration. *p* ≤ 0.05 (*) and *p* ≤ 0.001 (***) were considered statistically significant (*n* = 2; replicates = 2).

After differentiation of the pre-adipocytes 3T3-L1 cell line according to the manufacturer’s instructions, adipocytes were treated with the selected concentrations (1.25 and 2.5 mg/mL) to evaluate lipidic accumulation. The data collected showed a significant accumulation of lipids following the differentiation of pre-adipocytes into adipocytes, and a significant reduction following treatment of the adipocytes with magnolol (positive control) and with a concentration of 1.25 mg/mL of the cosmeceutical product (Figure 7).

## 3. Discussion

In the last few years, interest in anti-ageing preparations or cosmeceuticals and their potential ability to enhance a person’s more youthful appearance has progressively increased. Anti-ageing topics, with their multiple claims, seemingly limitless key active ingredients and complex formulations are leading the way during this huge period of growth in the cosmeceuticals industry, especially since the population is increasingly opting for less invasive, non-surgical alternatives to slow the effects of ageing on the skin [21]. The challenge for manufacturers is to create new skin products that contain a mix of active ingredients able to act in synergy on different anti-ageing targets while ensuring safety and providing a benefit against physiological skin blemishes. Some blends can be aggressive towards the most sensitive skin types or can promote the phenomenon of cosmetic acne in oily skin [15].

Working on a formula that can be used on different skin typologies while maintaining its effectiveness without side effects is another regarded aspect in cosmetology. In our study, the safety and effectiveness of a booster lotion (PHA TOUCH, Matex Lab Spa, Brindisi, Italy) enriched with different functional molecules, such as gluconolactone, mandelic acids, salicylic acids, phytic acids and different peptides, were investigated. Starting from the scientific evidence related to the intrinsic properties of these compounds, our study was conducted in parallel on the main cell lines (keratinocytes, fibroblasts, adipocytes and sebocytes) on which they exert their activity. The first step was to test cell viability and biosafety on each cell line, in order to select the first non-cytotoxic concentrations of the booster lotion to perform the following investigations. The collected results showed that, after 24 h of treatment, 1.25 and 2.5 mg/mL were the first two non-cytotoxic concentrations for keratinocytes (HaCaT), fibroblasts (NHDF) and adipocytes (3T3-L1). The fourth line of sebocytes (PCi-SEB_CAU) proved to be more sensitive in a 12-well plate cell viability assay (verified before performing mRNA expression analysis) and, even if the preliminary MTT assay in a 96-well plate shows an absence of cytotoxicity at all concentrations tested, 0.156 and 0.313 mg/mL were the concentrations selected for the efficacy investigation on this cell line. The potential risk of skin irritation was evaluated on a reconstructed human epidermis (RHE) 3D model following the referenced standard methods [26,27]. The results showed a viability of 105% and an amount of interleukin IL-1α less than 10 pg/mL (the threshold to identify a substance as an irritant), confirming the biocompatibility of the skin care lotion. The two concentrations (1.25 and 2.5 mg/mL) of the product selected by the preliminary MTT test on NHDF cells were chosen to investigate collagen and elastin stimulation. The obtained results showed a significant increase in both of the structural proteins involved in extracellular matrix (ECM) organization. Since their stimulation contributes to a rejuvenated appearance, including greater skin elasticity, plumpness and smoothness, the dermocosmetic’s ability to counteract skin ageing was demonstrated. The stimulation of these matrix components also influences pore minimizing and skin texture [10].

Furthermore, the pores ageing-related dilatation is associated to two other molecular processes: the parakeratosis status due to an accelerated and incomplete keratinization of keratinocytes into corneocytes during the differentiation and the presence of abundant unsaturated fatty acids, such as oleic acid in the sebum. To restore the normal keratinization process and thus reduce the unsightly appearance of the dilated pores, it is necessary to stimulate the differentiation of the keratinocytes, favoring the renewal of the nucleated cells around the dilated pores by new mature corneocytes [12,13]. In order to evaluate the ability of the booster lotion to stimulate the keratinocytes differentiation, HaCaT cells were treated for different time points (24-48-72 h), with the concentrations selected by preliminary MTT (1.25–2.5 mg/mL); a quantification of mRNA expression levels of the involucrin (IVL) was also performed. Involucrin is a protein component of human skin and, in humans, is encoded by the IVL gene. In association with the loricrin, it contributes to the formation of a cell envelope that protects corneocytes in the skin.

Therefore, the increase in IVL levels can be a valid means by which to study the cellular differentiation of keratinocytes [28]. The obtained data highlighted that the expression of the IVL differentiation marker significantly increases compared to untreated cells after 24 h of treatment with the cosmetic product at a concentration of 2.5 mg/mL. Therefore, in addition to the data obtained on collagen and elastin, the stimulation of keratinocyte differentiation strengthens the effectiveness of a formulation for smoothing and refining pores. The last molecular process related to the pore’s dilatation concerns the production of sebum and its control. In our study plan, two molecular targets involved in sebum production and in the lipogenesis process were selected and investigated in parallel in order to study the effect of the lotion on the baseline production of sebum and the potential stimulation of the same, which could give the product comedogenic properties. Sebocytes are the predominating cells around sebaceous glands, and they express dihydrotestosterone (DHT) receptors, which influence sebaceous gland activity in sebum secretion. The steroid 5-alpha reductase gene (SRD5A) plays a key role in the regulation of DHT; thus, the inhibition of its expression can decrease the amount of DHT in cells, leading to facial sebum control [11]. Data obtained from sebocytes (PCi-SEB_CAU) treatment with the product at concentrations of 0.156 and 0.313 mg/mL, selected by a second confirmatory MTT in a 12-well plate, highlighted that treatment with the cosmeceutical at all tested concentrations did not determine a variation of SRD5A3 gene expression compared to the untreated cells, proving that the product does not affect sebaceous lipid production and, therefore, can be considered non-comedogenic. The peroxisome proliferator-activated receptor gamma (PPARγ) is mostly found in the more differentiated suprabasal keratinocytes, in skin adipocytes and sebaceous glands (SGs), where it plays a critical role in differentiation and lipid formation [16]. The results collected regarding PPARγ gene expression in the adipocytes cell line (3T3-L1) after treatment with the selected concentration of the product (1.25 and 2.5 mg/mL) showed that treatment with both concentrations tested led to a statistically significant reduction in the marker compared to the untreated cells, suggesting a decrease in the adipogenesis and lipogenesis processes. Lipid accumulation was also investigated with Oil Red O staining in pre-adipocytes, adipocytes after differentiation, adipocytes treated with the selected concentration of the product (1.25 and 2.5 mg/mL) and adipocytes treated with magnolol (as the positive control) [29]. The results obtained showed a significant reduction in lipid accumulation following the treatment of the adipocytes with a concentration of 1.25 mg/mL of the cosmeceutical product. The data obtained on sebocytes and adipocytes cell lines demonstrated the non-comedogenic property of the formulation and, in particular, its influence on lipogenesis and sebum control, with a consequent beneficial effect of minimizing pores.

Lastly, the anti-photo ageing activity of the lotion was investigated in a model of stress-induced premature senescence of NHDF cells exposed to UVB irradiation as the source of extrinsic damage. The ability of the PHA to penetrate the dermis protecting fibroblasts from UV damage has been proven in several scientific works [30]. NHDF cells were treated with 1.25 and 2.5 mg/mL of the product and irradiated with 38 mJ/cm^2^ to evaluate their potential ability to prevent cells from photo-ageing by β-galactosidase staining. Skin ageing is closely related to cellular senescence, characterized by complex changes in the cell’s protein expression profile, which lead to replicative arrest and cellular dysfunction [31]. In particular, senescent cells are characterized by a strong increase in the secretion of growth factors, inflammatory cytokines and proteolytic enzymes, as well as an increase in the activity of the pH-dependent β-galactosidase defined; for this reason, β-galactosidase is associated with senescence (SA-βgal). The premature senescence of cells can be induced by exposure to a variety of oxidative stresses, such as DNA damage agents [32]. Therefore, UVB radiation can be used to create a model of stress-induced premature senescence of human dermal fibroblasts as an alternative in vitro model to study the potential anti-photo ageing protective effects of cosmetic products. The results obtained showed a statistically significant reduction in SA-βgal activity levels of 54% and 44%, respectively, after treatment with two selected concentrations of 1.25 and 2.5 mg/mL, demonstrating the anti-photo ageing effect of the formula.

## 4. Materials and Methods

### 4.1. International Nomenclature of Cosmetic Ingredients (INCI)

The functional classification of ingredients contained in the tested cosmeceutical is summarized in Table 1.

### 4.2. Cell Cultures

Different monolayer cell lines and a 3D model were used to perform the safety and efficacy assays. The cell lines were human keratinocytes (HaCaT, BS code CL 168), provided by I.Z.L.E.R. (Zooprofilattico Institute of Lombardy and Emilia Romagna, Brescia, Italy); normal human dermal fibroblasts (NHDF-Ad-Der Fibroblasts, code CC-2511, Lonza, Basel, Switzerland); sebocytes (PCi-SEB_CAU, Phenocell, Grasse, France) were developed from human induced pluripotent stem cells (iPSC); 3T3-L1 pre-adipocytes (SP-L1-F, Zen-Bio, Durham, NC, USA), derived from mouse embryo tissue. The 3D model was a reconstructed human epidermis (RHE) (Episkin Laboratories, Lyon, France). HaCaT cells were grown in a complete medium constituted of dulbecco’s modified eagle’s medium (DMEM; Euroclone, Milan, Italy) supplemented with fetal bovine serum (FBS; Euroclone, Milan, Italy), 1 mM L-glutamine (Capricorn Scientific, Ebsdorfergrund, Germany) and antibiotics (10,000 U/mL penicillin G and 10 mg/mL streptomycin sulfate (Euroclone, Milan, Italy). NHDF cells were grown in fibroblast basal medium (FBM, Lonza, Basel, Switzerland) supplemented with FGM-2 SingleQuot kit Supplement and Growth Factors (Lonza, Basel, Switzerland). PCi-SEB_CAU were grown in PhenoCULT-SEB sebocytes basal culture medium with supplement A (Phenocell, Grasse, France). Furthermore, 3T3-L1 pre-adipocytes were cultured in 3T3-L1 pre-adipocytes medium (Zen-Bio, Durham, NC, USA); two days after reaching confluence, the culture medium was switched to differentiation medium (DM-2-L1, Zen-Bio, Durham, NC, USA) for 3 days at 37 °C. Then, cells were maintained in maintenance medium (AM-1-L1, Zen-Bio, Durham, NC, USA) that was replaced every 2 days. Each cell line was grown in conditions of complete sterility and maintained in humidified atmosphere incubation at 37 °C with an atmosphere of 5% carbon dioxide (CO_2_). For the RHE model, two different media were used for maintenance and growth (Episkin Laboratories, Lyon, France).

### 4.3. Cell Viability Assay

Cell viability was determined by 3-(4,5-dimethylthiazol-2-yl)-2,5-diphenyl tetrazolium bromide) (MTT), a tetrazolium salt used to test cell proliferation and viability based on mitochondrial efficiency [33]. Briefly, for the preparation of the assay, cells were seeded (2 × 10^4^ for HaCaT, 1.7 × 10^4^ for NHDF, 1 × 10^4^ for adipocytes and 1 × 10^5^ for sebocytes experiments) into 96-well plates. After 24 h, cells were treated with the tested product at a 2.5 mg/mL concentration and the following (1:2) dilutions (tested range 0.156–2.5 mg/mL) were dissolved and vortexed in complete culture medium; untreated cells were used as the control. After 24 h of treatment, the culture medium was discarded and the cells were incubated with the MTT (SIGMA-ALDRICH, St. Louis, MO, USA) solution (1 mg/mL) at 37 °C for 2 h. Then, the solution was removed and replaced with 100 µL of dimethyl sulfoxide (DMSO, Honeywell, NC, USA). The absorbance was read at a wavelength of 560 nm using a microplate reader (Glomax Discover, Promega, Madison, WI, USA). Cell survival was calculated by measuring the difference in the optical density (OD) of the tested product with respect to the control (untreated cells) (*n* = 2; replicates = 3) [33].

Cell viability (%) = [OD_570_ nm test product/OD5_70_ nm control] × 100 (1)

### 4.4. Evaluation of Skin Irritation on the Reconstructed Human Epidermis (RHE) 3D Model

The potential skin irritation of the cosmeceutical product was evaluated on a RHE 3D model (SkinEthic, Nice, France), according to DB-ALM Protocol n°135 [26]. RHE inserts were placed in a maintenance medium (6-well plate) under sterile conditions and incubated at 37 °C, 5% CO_2_ overnight. After 24 h, the lotion was applied in toto on the surface of the epithelium insert for 42 min at 32 μL/cm^2^ concentration.

Negative (DPBS) and positive controls, consisting of a 5% *w*/*v* solution of SDS (Sigma-Aldrich, St. Louis, MO, USA) representing the irritating treatment, were also performed. At the end of the treatment, RHE inserts were rinsed with DPBS (25×) and then transferred into 6-well plates for incubation in a 37 °C, 5% CO_2_, 95% humidified atmosphere for 42 h. After this incubation time, the treated inserts were transferred into a 24-well plate filled with 300 µL of MTT solution (1 mg/mL), prepared according to the manufacturer’s instructions and incubated for a further 3 h at 37 °C, 5% CO_2_. Formazan crystals were dissolved in isopropanol (VWR Chemicals, Milan, Italy), and the OD of the samples was obtained by spectrophotometry at a 570 nm wavelength using a microplate reader. The data were elaborated as a ratio of the corrected optical densities of the sample over the negative control (sample treated with DPBS), where cell viability values ≤50% are an index of irritation:

Cell viability (%) = [OD_570 nm_ test product/OD_570 nm_ negative control] × 100 (2)

To better evaluate the skin irritation effect of the cosmetic cream, as well as the viability resulting from the direct contact of the product on the insert’s surface, the levels of interleukin (IL)-1α released after treatment were also measured after a recovery time of 42 h using an ELISA kit (Invitrogen, Waltham, MA, USA) following the manufacturer’s instructions. The absorbance was then measured at 450 nm using a microplate reader, and the IL-1α quantification was obtained by plotting the mean absorbance of each sample with a 5-PL standard curve (1.6–1000 pg/mL). IL-1α values ≥ 10 pg/mL are considered an indicator of irritation [27].

### 4.5. Evaluation of Collagen and Elastin Synthesis in NHDF Cells

The ability of the cosmeceutical product to modulate collagen production was evaluated in NHDF cells after 24-h treatment by quantitative Real-Time Polymerase Chain Reaction (qRT-PCR) assay. NHDF cells were homogeneously seeded in a 12-well plate at a density of 1.8 × 10^5^ cells/well. After 24 h, cells were treated with the first concentrations of the product proven to be non-cytotoxic and with the best solubility in the medium (1.25 and 2.5 mg/mL). Untreated cells were used as the control. After 24 h of treatment, total RNA was extracted from NHDF cells using the RNeasy Mini Kit (Qiagen, Hilden, Germany) and quantified using Multiskan and uDrop Duo Plate (Thermo Scientific, Waltham, MA, USA). Subsequently, total RNA was reverse transcribed into complementary DNA (cDNA) using an iScript™ Reverse Transcription Supermix kit (Bio-Rad, Hercules, CA, USA). PCR amplifications were performed using a SsoAdvanced Universal SYBR Green Supermix (Bio-Rad, Hercules, CA, USA); collagen type1, α1 (COL1A1) and elastin (ELN) were selected as target genes and gliceraldehyde-3-phosphate dehydrogenase (GAPDH) to normalize gene expression levels using the 2^−ΔΔCt^ method. The tool used for the analysis was the CFX Connect (Bio-Rad, Hercules, CA, USA) and the data were analyzed using CFX Maestro 1.1. software (version n° 4.1.2433.1219, Bio-Rad, Hercules, CA, USA).

### 4.6. Evaluation of the Effect on Keratinocytes Differentiation

To assess the effect of the product on the gene expression of keratinocyte differentiation, involucrin (IVL) was selected as the biomarker. HaCaT cells were homogeneously seeded in a 12-well plate at a density of 2 × 10^5^ cells/well and incubated at 37 °C, with a 5% CO^2^ humidified atmosphere. After 24 h, the cells were treated with the tested cosmetic product at a concentration of 1.25–2.5 mg/mL selected after the preliminary MTT. Non-treated cells were used as the control, while cells treated with a high calcium concentration (Ca^2+^ 1.8 mM) were used as the positive control. The effect on keratinocytes differentiation was investigated after 24–48 and 72 h of treatment; at each time point, the cells were harvested for RNA extraction using an RNeasy Mini Kit (Qiagen, Hilden, Germany). RNA quantification was performed using Multiskan and uDrop Duo Plate (Thermo Scientific, Waltham, MA, USA). Retro-transcription of toral RNA in cDNA was performed using an iScript™ Reverse Transcription Supermix kit (Bio-Rad, Hercules, CA, USA). PCR amplifications were performed using a SsoAdvanced Universal SYBR Green Supermix (Bio-Rad, Hercules, CA, USA); GAPDH was used as a housekeeping gene to normalize gene expression using the 2^−ΔΔCt^ method. The tool used for the analysis was the CFX Connect (Bio-Rad, Hercules, CA, USA) and the data were analyzed using CFX Maestro 1.1. software (version n° 4.1.2433.1219, Bio-Rad. Hercules, CA, USA).

### 4.7. Evaluation of Anti Photo-Ageing Effect on NHDF-Irradiated Model

The premature senescence of cells can be induced by exposure to a variety of oxidative stresses, such as DNA-damaging agents. Therefore, UVB radiation was used to create a model of the stress-induced premature senescence of NHDF cells, in order to study the potential anti-photo ageing protective effects of cosmetic products. First, a preliminary investigation on cell viability was conducted to identify the first concentrations non-cytotoxicity in association with the UVB dose selected (38 mJ/cm^2^). NHDF cells were homogeneously seeded in a 12-well plate at a density of 1 × 10^5^ cells/well and incubated at 37 °C in a 5% CO_2_ humidified atmosphere. After 24 h of incubation, cells were treated with 1.25 and 2.5 mg/mL of the product and re-incubated at 37 °C in a 5% CO^2^ humidified atmosphere. After the treatment time (24 h), cells were washed with DPBS (Sigma-Aldrich, St. Louis, MO, USA), irradiated with UVB (38 mJ/cm^2^) using a UVB lamp (CAMAG UV lamp 4 dual 302 wavelength, Camag, Grosshöchstetten, Switzerland) and incubated at 37 °C with 5% CO^2^ for another 24 h. Untreated cells were used as the control (Ctrl). The day after the MTT protocol was performed, 1.25 and 2.5 mg/mL were also confirmed in association with the 38 mJ/cm^2^ UVB dose to evaluate the effect of the product to prevent cells from photo-ageing using β-galactosidase colorimetric assay. The assay was conducted using the Senescence Cells Histochemical Staining kit (Sigma-Aldrich St. Louis, MO, USA). Briefly, NHDF cells were homogeneously seeded in 12-well plates and then treated for 24 h with non-cytotoxic concentrations of the tested product (1.25 and 2.5 mg/mL). After treatment, cells were washed with DPBS and irradiated with 38 mJ/cm^2^. After 24 h of recovery and two washes in DPBS, 400 µL of Fixative Buffer provided by the kit were added to each well at room temperature. Finally, after adding the Staining Mixture (prepared according to the manufacturer’s instructions), NHDF cells were then incubated at 37 °C overnight and analyzed under a light inverted microscope (VisiScope IT415 PH, VWR, Radnor, PA, USA). The following day, images were acquired (Camera, 5 plus, 5MP, Moticam, Barcelona, Spain) at 20× magnification, and total and stained cells were counted (at least 1000 cells per condition), calculating the percentage of positive cells as a sign of senescence.

### 4.8. Evaluation of Sebum Production and Adipogenesis

The expression of markers involved in sebum production and adipogenesis were evaluated, respectively, in sebocytes (PCi-SEB_CAU) and adipocytes (3T3-L1) after 24 h of treatment with the cosmeceutical using the RT-qPCR technique. Untreated cells were used as the control. To perform this investigation, cells were homogeneously seeded in a 12-well plate and incubated at 37 °C, with a 5% CO_2_ humidified atmosphere. After 24 h, the cells were treated with the tested cosmetic product at concentrations of 0.156 and 0.313 mg/mL for sebocytes and 1.25 and 2.5 mg/mL for adipocytes. The treatment concentrations were selected following a second MTT performed in a 12-well plate. Data on adipocytes, on the other hand, confirmed the cytotoxicity tests performed in 96-well plates. Therefore, after 24 h of treatment, cells were harvested for RNA extraction using the RNeasy Mini Kit Kit (Qiagen, Hilden, Germany). RNA quantification was performed using Multiskan and uDrop Duo Plate (Thermo Scientific, Waltham, MA, USA). Retro-transcription of toral RNA in cDNA was performed using iScript™ Reverse Transcription Supermix kit (Bio-Rad, Hercules, CA, USA). PCR amplifications were performed using a SsoAdvanced Universal SYBR Green Supermix (Bio-Rad, Hercules, CA, USA). Real-time PCR analysis was performed on the target gene steroid-5αreductase-3 (SRD5A3) for the evaluation of sebum production and peroxisome proliferator activated receptor γ (PPARγ) for the evaluation of adipogenesis. GAPDH was used as a housekeeping gene to normalize gene expression of SRD5A3, and Actin-β (ACTB) was used as a reference gene to normalize PPARγ. The tool used for the analysis was CFX Connect (Bio-Rad, Hercules, CA, USA) and the data were analyzed using CFX Maestro software (Bio-Rad. Hercules, CA, USA).

The lipid content in differentiated adipocytes was also investigated by oil red staining. Pre-adipocytes were seeded in a 12-well plate until confluence, then an internal differentiation protocol was carried out. Cells were treated with non-cytotoxic concentrations of the product resulting from preliminary MTT assay (1.25 and 2.5 mg/mL) and with 20 µM of magnolol (Sigma-Aldrich, St. Louis, MO, USA) as the positive control. An undifferentiated control and a control of differentiated and untreated cells were maintained. At the end of the treatment, the Oil Red O kit (Sigma-Aldrich, Louis, MO, USA) was used to stain the cells. The samples were fixed with formalin provided by the kit, washed twice with water and subsequently stained with Oil Red O solution for 10–20 min and haematoxylin for 1 min. The lipid drops were viewed under a light inverted microscope (VisiScope IT415 PH, VWR, Radnor, PA, USA) and the images were acquired with a camera (Camera 5 plus, 5MP, Moticam, Barcelona, Spain) at 20× magnification; lipid accumulation was quantified using a microplate reader (Multiskan, Thermo Scientific, Waltham, MA, USA) at a wavelength of 492 nm after extraction with isopropanol (VWR Chemicals, Milan, Italy).

### 4.9. Statistical Analysis

Results were presented as mean ± SD of independent experiments performed at least in duplicate. Statistical significance was calculated using one-way ANOVA followed by Fisher’s LSD test as a post-hoc test. Values of *p* < 0.05 were considered statistically significant compared to the relative controls. Statistical analyses were performed using GraphPad Prism version 9.0.0 (GraphPad Software Inc., San Diego, CA, USA).

## 5. Conclusions

Today, cosmeceutical marketing is expanding faster than empirical evidence of efficacy and safety can be acquired. To fill these gaps, it is essential to understand the theoretical principles and develop innovative studies that can allow for the rapid evaluation of these complex formulas [34,35,36]. Collectively, our study provides a valid experimental plan to prove the biocompatibility and safety of the booster lotion under study through a series of in vitro tests. The data collected also proved its non-comedogenic properties, as well as its anti-photo ageing effect related to its ability to reduce the number of senescent cells after UV damage. The anti-ageing effect was studied on the main markers of ageing specifically related to the imperfection of dilated pores typical of subjects over 40 years of age. The stimulation of keratinocyte differentiation, collagen and elastin levels, together with the control of sebum production, demonstrate the efficacy of the enriched lotion to smooth/refine pores. Therefore, the chosen in vitro experimental models are an adequate tool to by which demonstrate the biosafety and efficacy of a complex formula enriched with active ingredients and can be of support for future, more in-depth in vivo studies on these ageing-related claims and beyond.

## Figures and Tables

**Table 1:** Functional classification of ingredients contained in the cosmetic used in the study.

Function	Ingredients
Humectant	Propanediol, Sodium Hyaluronate, Xylityl Sesquicaprylate, Acetyl Heptapeptide-4
Emollient	Caprylic Glycol,
Emulsifier	Xylityl Sesquicaprylate
Solvent	Aqua, 1,2-Hexanediol
pH regulator	Sodium Hydroxide
Denaturant	Sodium Hydroxide
Antimicrobic	Mandelic Acid, Xylityl Sesquicaprylate, Phenoxyethanol
Skin conditioning	Gluconolactone, Salycilic Acid, Sodium Hyaluronate, 1,2-Hexanediol, Acetyl Heptapeptide-4, Acetyl Tetrapeptide-40, Xylityl Sesquicaprylate, Caprylic Glycol, Caesalpina spinosa gum
Preservative & Perfume	Salycilic Acid, Xylityl Sesquicaprylate, Caprylic Glycol, Phenoxyethanol
Chelating Agent	Gluconolactone, Phytic Acid, Caprylhydroxamic Acid,
Surfactant	Heptyl Glucoside, Xylityl Sesquicaprylate
Viscosity controlling	Ammonium Acryloyldimethyltaurate/VP Copolymer, Caesalpina spinosa gum
Keratolytic	Salycilic Acid
Film forming	Caesalpina spinosa gum

## Data Availability

Data is contained within the article; raw data are available from the corresponding author.
